# Individual Responses versus Aggregate Group-Level Results: Examining the Strength of Evidence for Growth Mindset Interventions on Academic Performance

**DOI:** 10.3390/jintelligence11060104

**Published:** 2023-05-30

**Authors:** Mariel K. Barnett, Brooke N. Macnamara

**Affiliations:** Department of Psychological Sciences, Case Western Reserve University, Cleveland, OH 44106, USA

**Keywords:** growth mindset interventions, academic performance, heterogeneity, persons as effect sizes, percent correct classification

## Abstract

Mindset theory assumes that students’ beliefs about their intelligence—whether these are fixed or can grow—affects students’ academic performance. Based on this assumption, mindset theorists have developed growth mindset interventions to teach students that their intelligence or another attribute can be developed, with the goal of improving academic outcomes. Though many papers have reported benefits from growth mindset interventions, others have reported no effects or even detrimental effects. Recently, proponents of mindset theory have called for a “heterogeneity revolution” to understand when growth mindset interventions are effective and when—and for whom—they are not. We sought to examine the whole picture of heterogeneity of treatment effects, including benefits, lack of impacts, and potential detriments of growth mindset interventions on academic performance. We used a recently proposed approach that considers persons as effect sizes; this approach can reveal individual-level heterogeneity often lost in aggregate data analyses. Across three papers, we find that this approach reveals substantial individual-level heterogeneity unobservable at the group level, with many students and teachers exhibiting mindset and performance outcomes that run counter to the authors’ claims. Understanding and reporting heterogeneity, including benefits, null effects, and detriments, will lead to better guidance for educators and policymakers considering the role of growth mindset interventions in schools.

## 1. Introduction

Students who do well in school are more likely to secure a stable career, reach financial success, and experience higher degrees of happiness than students with lower academic achievement (e.g., National Association of Colleges and Employers 2019; Quinn and Duckworth 2007; Rose and Betts 2004). Unsurprisingly, many people are invested in improving students’ academic performance. Parents aim to foster success in their children, educators work to support pupils’ achievement in the classroom, and policymakers pursue funding for interventions that improve student outcomes.

By aiming to foster improved academic performance and promote achievement, one idea in particular has gained massive popularity in schools: mindset theory (i.e., implicit theories; Dweck 2000). According to mindset theory, “what students believe about their brains—whether they see their intelligence as something that is fixed or something that can grow and change—has profound effects on their motivation, learning, and school achievement” (Dweck 2008, p. 110). That is, students who see their intelligence or another attribute (e.g., personality) as fixed, tend to focus on appearing smart rather than on learning, avoid effort when challenged, and give up when faced with a setback. In contrast, students who see their intelligence or another attribute as something that can grow and change, are eager to learn, work hard when challenged, and persevere when facing a setback (Rattan et al. 2015). Given that these traits and behaviors are assumed to be important for student success, it is unsurprising that 88% of teachers in the U.S. believe the use of growth mindsets with students is important for student academic outcomes (Yettick et al. 2016).

A small industry offering growth mindset interventions has flourished in recent years. The interventions typically aim to teach a growth mindset by explaining the concept through reading, presentation, or an interactive game (Sisk et al. 2018). For example, MindsetWorks, LLC, sells a growth mindset intervention computer program, “Brainology”, that teaches students that intelligence can be developed with effort using lessons, online reflections, and activities. Mindset’s popularity has been described as a “revolution that is reshaping education” (Boaler 2013, p. 143), with growth mindset interventions being implemented in classrooms around the world (Sisk et al. 2018).

### 1.1. Are Academic Growth Mindset Interventions Effective? A High-Level Lens

Recently, two meta-analyses were published examining the efficacy of growth mindset interventions. One (Macnamara and Burgoyne 2022) reported effects on academic performance and how studies’ adherence to best practices in study design, reporting, and avoiding bias might influence the size of those effects. They also examined a large number of theoretical and methodological moderators. No theoretical moderators yielded significant effects, and model effects were null once best practices in study design, reporting, and avoiding bias were taken into account. Macnamara and Burgoyne (2022) concluded that the effect of growth mindset interventions on academic performance might be rare if not spurious. 

The other meta-analysis (Burnette et al. 2022) reported effects on multiple outcomes, one of which was academic performance. They focused on two moderators: implementation fidelity and “focal group” status, where focal groups were identified by the original study authors as subgroups expected to benefit most from the growth mindset intervention. These focal groups ranged in their characteristics from students with fixed mindsets, to students with low grades, to students from ethnic minority backgrounds. The academic performance effect size for studies with high implementation fidelity on their targeted focal groups was $\bar{d}$ = 0.14; for non-focal groups, the effect was considerably smaller, $\bar{d}$ = 0.04. Burnette et al. (2022) emphasized the need to examine the heterogeneity of treatment effects. They concluded that while positive effects should be expected under some circumstances, that “null and even negative (in the case of academic achievement) effects are [also] to be expected in growth mindset interventions” (p. 27). We discuss the heterogeneity of treatment effects in further detail below. 

### 1.2. The “Heterogeneity Revolution”

Heterogeneity of effects has become an avenue of interest as of late, and there are many different forms of heterogeneity. The one most often discussed in the growth mindset literature is the heterogeneity of intervention effects, in which researchers explore whether certain groups respond to the intervention to a greater degree than other groups. Beyond differences in treatment effects corresponding to different student characteristics, researchers from educational and organizational settings (e.g., Domitrovich et al. 2008; Klein and Sorra 1996) have also advocated for considering differences in implementation quality at the intervention and the support levels, which might also account for the heterogeneity of effects. 

A new argument among mindset proponents is that researchers should expect heterogeneity in treatment effects because of sample characteristics (i.e., at-risk groups; Burnette et al. 2022; Tipton et al. 2023). That is, rather than making claims about large gains in student achievement and performance outcomes overall (e.g., Dweck 2008), the focus has shifted to groups and circumstances where growth mindset interventions appear most effective (Bryan et al. 2021; Yeager and Dweck 2020; Yeager et al. 2019). For example, mindset researchers have called for a “heterogeneity revolution” in behavioral science (Bryan et al. 2021). In their call, Bryan et al. (2021) suggest that failures to replicate in psychology are due to failures to recognize heterogeneity in treatment effects. They propose that systematic examinations of heterogeneity will lead to more comprehensive theories, dependable guidance to policymakers, and a generalizable science of human behavior that will change the world. 

In support of the need to examine heterogeneity, Bryan et al. (2021) described the varying effects of the National Study of Learning Mindsets, a large-scale growth mindset intervention. In the first report of the National Study of Learning Mindsets, the authors focused on students performing below their school’s median performance (Yeager et al. 2019) and found a significant effect for this subgroup. A follow-up report later included the whole sample from this study but focused on treatment effects depending on students’ math teachers’ mindsets. When students’ math teachers had relatively higher growth mindsets, they found positive treatment effects on students’ academic achievement, but not when their math teachers had relatively lower growth mindsets (Yeager et al. 2022). The different effects of the National Study of Learning Mindsets, depending on how the researchers selected subsamples and measures from the same study, served as a demonstration of the importance of examining heterogeneity (Bryan et al. 2021). 

In addition to the National Study of Learning Mindsets, Bryan et al. (2021) also described a hypothetical growth mindset intervention. They illustrate that, depending on whether one examines the whole sample or particular subsamples, the treatment effect ranges from null to moderately positive. They argue that researchers should capitalize on heterogeneity to deepen our theoretical understanding and to improve interventions.

These recent calls to examine heterogeneity, particularly of treatment effects, provide examples of effects for some groups and no effects for others. However, more examinations of heterogeneity that account for the full range of treatment effects, such as individuals in subgroups who may exhibit detrimental outcomes, would be valuable for understanding the extent of treatment effect variability in academic performance. Here, we focus on individual-level variation in treatment effects in terms of observed numerical benefits, no observed numerical impact, and observed numerical detriments to academic performance.

### 1.3. Examining the Whole Picture of Heterogeneity

We agree that there is a need to examine heterogeneity, particularly in growth mindset intervention studies. In line with Burnette et al.’s (2022) conclusion that positive, null, and negative effects are to be expected, we believe the whole range of effects needs to be considered. It is important to know how many students see positive impacts, how many students see no impact, and how many students see negative impacts from a given treatment. Aggregate results alone, consisting of group-level performance, do not capture the full scope of intervention effects varying from person to person. That is, statistical inferences and effect sizes for a group of individuals may not accurately describe how the individuals within the group responded to the treatment (Grice et al. 2020; Lamiell 2013; Molenaar 2004).

#### Persons as Effect Sizes

In addition to reporting inferential statistics and group-level effect sizes (e.g., *d*, η^2^), individual responses and behaviors can be quantified as effect sizes. How might this work? A recent paper (Grice et al. 2020) described the process of examining individual-level data to determine how many participants in a study behaved or responded consistently with theoretical expectation. The answer can be computed as a percentage. For example, if a researcher reports that 85% of participants responded according to expectations, this evidence is stronger than if the researcher had reported that only 45% of participants responded according to expectations. This straightforward percentage can be easily understood as an effect size by scientists, policymakers, and laypeople alike. 

Persons as effect sizes (Grice et al. 2020) is an approach that better describes individual responses than aggregate analyses. Grice et al. (2020) provide several case studies as demonstrations. In one example, Siegel et al. (2018) conducted a study where they hypothesized that presenting a face with positive, neutral, or negative affect would influence their ratings of a paired neutral face. In accordance with their hypothesis, Siegel et al. found a significant main effect of affect condition on mean rating, *p* < 0.001, a Bayes factor of 62.9, and a η^2^ = of 0.32. Pairwise differences between the three conditions were also significant in the hypothesized directions: positive > neutral > negative. However, when examining the individual-level data, Grice et al. found that only 11 of 45 participants, or 24.44%, matched the hypothesized pattern. The majority of participants rated faces differently than the expected positive > neutral > negative rating. The picture painted by individual-level patterns countered the conclusions, based on aggregate analyses, that had previously been made. 

The persons as effect sizes approach better represents the performance of individuals within a group than aggregate effect sizes, which may poorly represent the individuals within the groups (Grice et al. 2020; Lamiell 2013; Molenaar 2004). Persons as effect sizes is not an inferential statistic. That is, the method has no bearing on statistical significance. Rather, this approach is descriptive and provides an additional effect size that captures how many participants behaved or responded in line with theory. Person-centered effect sizes, in conjunction with aggregate effect sizes, can present a more complete picture of the results.

### 1.4. The Present Study

Here, we evaluate growth mindset intervention studies that allow for further understanding of individual-level heterogeneity of a treatment effect. As Grice et al. (2020) note, traditional measures of effect size allow researchers to answer questions about intervention-based differences and changes at the group level, but do not provide information as to the number of individuals who behaved consistently with theoretical expectation. By examining individual-level responses, researchers can examine previously overlooked patterns in the data that can provide information for fine-tuning a theory. Using individual-level descriptive analyses, we examine three papers that had previously tested and presented claims using aggregate group-level analyses about the benefits of growth mindsets on academic performance. 

In the present study, we evaluate individual mindset and academic performance outcomes associated with growth mindset interventions by extending the persons as effect sizes method developed by Grice et al. (2020). The higher the percentages of individuals within samples who behave or perform in line with theoretical expectations, the stronger the evidence for the original papers’ claims that growth mindset interventions change mindsets and/or are beneficial for students’ academic performance. According to Grice et al. (2020), 50% of participants responding in line with theory is expected due to chance. To illustrate, imagine a very large group of students whom we randomly divide into two groups. Neither group receives a treatment. In this scenario, on average, we should not observe any differences between the two groups. If we select a student from Group 1 and another student from Group 2, it should be a 50/50 chance that the student in Group 1 has a higher grade than the student in Group 2 and vice versa. Thus, if the percentage of participants responding according to theoretical expectation hovers around 50%, the results are not very impressive, as the probability of participants responding in line with theory is equal to that of the probability of participants responding inconsistently with expectation. 

The persons as effect sizes approach can be expanded to describe the full extent of heterogeneity. Rather than only determining the number of participants who behaved or responded consistently with theory (e.g., treatment students who numerically outperformed their control-group counterparts, treatment students whose grades numerically improved), as in Grice et al.’s work (Grice et al. 2020), we can also calculate the number of participants who experienced no change in their behavior or response (e.g., treatment students with identical grades to their control-group counterparts, treatment students with identical pre- to post-treatment grades), and the number of participants who responded or behaved opposite to the theory (e.g., treatment students who performed numerically worse than their control-group counterparts, treatment students whose grades numerically decreased). This approach provides insights for developing a complete theory by examining variation in individual responses that might otherwise be lost in an aggregate approach. We therefore extend Grice et al.’s (2020) approach. Rather than presenting a single percentage, the percentage of those who responded according to expectations, and treating ties and those responding counter to expectations as the remainder, we separately report ties and the percentage of those responding opposite of the study claims. Thus, we present three percentages for each research question: the percent who behaved or responded according to study claims (i.e., Grice et al.’s 2020, percent correct classification), the percent who exhibit no numerical difference in their response, and the percent who behaved or responded counter to study claims.

Examining the percentage of students who behave in line with, who demonstrate no difference, and who behave counter to expectations is necessary for understanding how well the aggregate effects represent the individuals in the sample. A significant effect with a given aggregate effect size can occur from multiple patterns of individual results, from only a few individuals behaving in line with expectations, to the majority of individuals exhibiting expected treatment effects. In analyzing individual and aggregate results jointly, evidence may offer novel implications about cost–benefit tradeoffs and about potential risks. Only by understanding the full range of outcomes, from benefits to detriments, can we develop complete theories and provide better guidance to parents, educators, and policymakers.

## 2. General Methods

### 2.1. Search

We searched for published papers claiming to provide evidence that growth mindset interventions were beneficial for academic performance with publicly posted individual-level data. We focused on published papers because those provide public claims for us to evaluate. We were interested in calculating persons as effect sizes for claims by the original study authors; unpublished studies range from having no associated text to evaluate to a completed draft where interpretations and claims could change prior to publication. 

We searched the open data of recently published meta-analyses on growth mindset interventions (Burnette et al. 2022; Macnamara and Burgoyne 2022). Macnamara and Burgoyne (2022) provided information in their open data about whether each of the original studies had open data. Only 2 of the 63 original studies in Macnamara and Burgoyne (2022) had associated open data (Ehrlinger et al. 2016; Yeager et al. 2016). We next searched the original studies included in Burnette et al. (2022)’s meta-analysis of academic performance. The two meta-analyses had different inclusion criteria and search stop dates and therefore did not perfectly overlap in the studies they included. We found one additional published study with open individual-level data. However, this study (Alan et al. 2019) was framed as a grit intervention, and the original study authors did not make any claims about growth mindset associated with their study results. Therefore, the study is not included here. We became aware of another growth mindset intervention study with individual-level data published following the meta-analyses’ stop search dates (Porter et al. 2022) and included it as well. 

We analyzed the results from these three growth mindset intervention studies. We know of no other published growth mindset intervention studies with an academic performance outcome that provides open individual-level data. That said, this was not a large-scale systematic search that would be conducted for a meta-analysis or systematic review. Instead, we relied on the data from two large-scale systematic searches associated with meta-analyses. Studies may exist that were missed in our search, especially studies in the last few years after the stop search dates of the meta-analyses. However, based on our search, published growth mindset intervention studies with individual-level open data appear rare; therefore, we are unlikely missing many studies. Relatedly, because such studies are rare, the three studies we evaluate are not necessarily representative of the literature for growth mindset interventions on academic performance. Future researchers may wish to conduct a systematic search that includes unpublished data. 

The three included studies differ substantially from one another. The first, Yeager et al. (2016), examines effects from a growth mindset of personality intervention on students’ grades. The second, Ehrlinger et al. (2016), examines effects from a laboratory growth mindset of intelligence intervention on students’ overconfidence and attention to easy and difficult problems on an academic achievement measure. The third, Porter et al. (2022), examines effects from a growth mindset of intelligence intervention on students’ and teachers’ mindsets, and on students’ grades. Thus, these studies are not comparable to one another. We do not seek to generalize these results, but to provide examples to evaluate the strength of the claims in this research area using the persons as effect sizes method. 

Open-source data for each of these studies can be found online via Open Science Framework: Yeager et al. (2016, https://osf.io/ufamv/, accessed on 15 July 2022), Ehrlinger et al. (2016, https://osf.io/fm5c2/, accessed on 15 July 2022), and Porter et al. (2022, https://osf.io/z2nvy/, accessed on 20 July 2022).

### 2.2. Claims

We evaluated claims about the effects of growth mindset interventions presented by the original study authors. We focused on claims presented in the abstract, highlights, or discussion sections. We note where the study authors’ claims conflicted with their own results or were unwarranted based on the study’s data. For each claim, we describe how we evaluated the strength of the evidence for the claim using the approach that considers persons as effect sizes.

### 2.3. Calculations

#### 2.3.1. Within-Subjects Comparisons

When original study authors made within-subjects comparison claims, we calculated within-subjects effect sizes. Within-subjects claims refer to changes (e.g., improvements) within individuals within a condition. An example of a within-subjects comparison claim is if original study authors implied that students who received the intervention experienced an increase in grades from pre- to post-intervention. 

To conduct within-subjects persons as effect sizes, we calculated the percentage of students who changed according to the claim. Continuing with the above example claim (students who received the intervention experienced an increase in grades from pre- to post-intervention), the effect size would be calculated by counting the number of students who received the intervention who experienced a numerical increase in grades from pre- to post-intervention, divided by the total number of students who received the intervention, then multiplied by 100 (number of treatment students whose grades changed as expected / total number of treatment students × 100). 

Differing from Grice et al.’s (2020) approach of reporting a single percentage of the participants who behaved or responded according to theory, we report three percentages: the percent who changed according to the claim, the percent where no numerical change was observed (e.g., identical pre- and post- grades), and the percent who changed counter to expectations (e.g., their grades numerically decreased). We additionally examined within-study claims (e.g., students receiving the intervention saw an increase in grades) by comparing change scores of treatment and control students using a between-subjects approach, described next. 

#### 2.3.2. Between-Subjects Comparisons

When original study authors made between-subjects comparison claims, we calculated between-subjects effect sizes. Between-subjects claims refer to comparisons between groups. An example of a between-subjects comparison claim is if original study authors claimed that students who received the intervention had better post-intervention grades than the control group. 

The between-subjects persons as effect sizes approach compares all pairs of individuals from the two groups. This technique is analogous to the Mann–Whitney U, except for how ties are treated. Continuing with the above example claim (students who received the intervention had better post-intervention grades than the control group), each possible treatment–control student pair is compared. The effect size is calculated by counting the number of pairs where the treatment student’s post-intervention grade was numerically higher than the control student’s in the pair, divided by the total number of treatment–control pairs, multiplied by 100 (number of pairs where the treatment student’s grade is higher than their control counterpart/total number of treatment–control student pairs × 100). To illustrate between-subjects comparisons concretely, suppose the authors of a hypothetical study administered an intervention to 50 students, had another 50 students as controls, and claimed that the students who received the intervention had higher post-intervention grades than the students in the control group. With this approach, we would examine each of the 2500 possible treatment–control pairs (50 × 50 = 2500) and calculate the percentage of treatment–control pairs where the treatment students’ grades were numerically higher than their control student counterpart.

When comparing two groups, a percent behaving according to theory of ~50% would be expected due to chance (Grice et al. 2020). If the intervention has no effect, then it is equally likely that the student receiving the intervention has better grades than the control student, as it would be that the control student has better grades than the intervention student. That is, around 50% of students in intervention will have better grades, and around 50% of control will have better grades. In contrast, if the intervention is effective, then more students in the intervention should have better grades than students in the control group. The larger the percentage of students who receive the intervention who outperform their control student counterparts, the greater the evidence of the effectiveness of the intervention, supporting the claims of the authors from the original study.

We also calculated the percent of treatment students with identical scores to their control student counterpart, and the percent of treatment students with lower scores than their control student counterpart. To calculate all between-subjects comparisons, we used the software package Observation Oriented Modeling (Grice 2020). 

## 3. Yeager et al. (2016) 

### 3.1. Introduction

Yeager et al. (2016) conducted two growth mindset (incremental theory) interventions with the aim of improving adolescent stress and coping during evaluative social situations. The intervention, a reading and writing exercise, lasted approximately 25 min and taught students that people do not have fixed personalities but instead have the potential to change their beliefs and motivations. Students in the active control condition received a reading and writing exercise of the same duration but focused on a topic unrelated to personality and adjustment (i.e., how areas of the brain help individuals adjust to new physical environments). In their first study, Yeager et al. (2016) evaluated the effect of a growth mindset intervention on Trier social stress test performance, threat appraisals, and cardiovascular and neuroendocrine responses. No measures of academic performance were included in this study; therefore, we do not consider study 1 here. 

In study 2, Yeager et al. (2016) administered the same approximately 25-min-long growth mindset intervention as in study 1 and measured grade point averages of 319 ninth grade students. The intervention was administered to ninth-graders in their first semester and grades were assessed at three time points: (1) pre-intervention, a composite of *z*-scored values for prior grades and test scores in core subjects; (2) post-intervention semester 1, a composite of grades in core subjects at the end of the semester that included the intervention; and (3) post-intervention semester 2, a composite of grades in core subjects at the end of the following semester. According to Yeager et al. (2016), “the incremental-theory manipulation improved grades up to 7 months after intervention” (p. 1089) and that “[s]tudents who received the intervention also had better grades over freshman year than those who did not” (p. 1078).

Yeager et al. made no other claims about intervention effectiveness on academic achievement in this study. Further, they did not make any claims about intervention effectiveness for specific subgroups. Readers should not confuse this study with another study authored by Yeager et al. in the same year, where they made specific claims about subgroups. That study does not have open student-level data associated with it and, therefore, cannot be included here.

### 3.2. Methods

Prior to analysis, we first needed to make several assumptions due to a lack of details in Yeager et al.’s (2016) methods. First, Yeager et al. do not state when the pre-intervention grades were assessed. We assume that prior grades are from the semester immediately prior to the semester in which the intervention was administered. Second, we are not sure why a *z*-scored composite of grades and test scores was used for prior grades, whereas a composite of only grades (no test scores and no mention of *z*-scoring) was used for post-intervention. Nonetheless, grades at each time point appeared to be on the same scale. Thus, we assume that prior grades are comparable to post-intervention grades. Third, Yeager et al. (2016) do not provide information as to when within the semester the intervention took place. Based on their claim that the intervention improved grades up to seven months later, we infer that the post-intervention semester 2 grades were assessed seven months after the intervention, as the first post-intervention time point was at the end of the same semester in which the intervention was administered, and semesters last approximately 4 months. 

We first sought to assess the strength of the evidence for the claim that “the incremental-theory manipulation improved grades up to 7 months after intervention” (p. 1089). We examined this within the context of both a within-subjects analysis, i.e., treatment students improved their grades, and a between-subjects analysis, i.e., relative to the control students, treatment students improved their grades more. 

There are three possible time point comparisons where change up to seven months post-intervention can be assessed. We first calculated comparisons from pre-intervention to post-intervention semester 1. This timeframe captures the change in grades from pre-intervention baseline, presumably the spring before the intervention, to the end of the semester following the intervention. This period is within the seven-month timeframe during which Yeager et al. claim to see improvements. That said, though Yeager et al. claim that the intervention improved grades up to seven months after the intervention, it may be that they meant the intervention improved grades only after seven months following the intervention. In this case, we would not expect to see impressive results for this period of pre-intervention to post-intervention semester 1.

Next, we compared pre-intervention to post-intervention semester 2 grades, the longest timeframe available. This timeframe may be the best assessment of Yeager et al.’s claim because it captures pre-intervention grades as well as grades seven months (we assume) after the intervention. Following, we compared post-intervention semester 1 to post-intervention semester 2 grades. If pre-intervention grades are not comparable (recall that pre-intervention achievement scores included test scores whereas post-intervention achievement scores did not), then this latter timeframe would offer the best test of Yeager et al.’s claim that grades improved up to 7 months after intervention. However, including pre-intervention (i.e., baseline) grades offers a better assessment of change due to the intervention. 

We also assessed the strength of the evidence for the claim that “[s]tudents who received the intervention also had better grades over freshman year than those who did not” (p. 1078), which is a between-subjects claim. We examined all possible treatment–control pairs at post-intervention semester 1 and semester 2.

We used pairwise deletion for missing values (e.g., a missing pre-intervention or semester grade) to retain as many data points as possible for change and comparison analyses.

### 3.3. Descriptive Results

Pre-intervention grades were a composite of course and test scores, which appeared to be on the same scale as post-intervention course grades; post-intervention grades were a composite of course grades. Pre-intervention grades ranged from 1.68 to 3.98; post-intervention semester 1 grades ranged from 1.25 to 4.00; and post-intervention semester 2 grades ranged from 1.00 to 4.00.

Yeager et al. (2016) reported that a total of 303 participants consented to complete the intervention and questionnaires, and to have their school records analyzed. However, Yeager et al. (2016, https://osf.io/fm5c2/ accessed on 15 July 2022) provide the pre-intervention grades for 316 students (intervention *n* = 160, control *n* = 156), the post-intervention semester 1 grades for 305 students (intervention *n* = 152, control *n* = 153), and the post-intervention semester 2 grades for 306 students (intervention *n* = 152, control *n* = 154).

### 3.4. Treatment Students’ Changes in Grades

We first examined students’ grades prior to and after the intervention. To assess the claim that the mindset manipulation improved grades up to seven months after the intervention, we first examined change in grades from pre-intervention to post-intervention semester 1 for the students who received the growth mindset intervention and had grades available at both time points. Table 1 provides the results of the percentage of treatment students who experienced improved grades (a numeric increase from pre-intervention to post-intervention), the percentage of students who experienced no change in grades (identical grades at both time points), and the percentage of students who experienced a decline in grades (a numeric decrease from pre-intervention to post-intervention). We repeated these calculations for pre-intervention to post-intervention semester 2, and for post-intervention semester 1 to post-intervention semester 2.

As can be seen in Table 1, the results from the analyses on treatment students’ changes in grades over time largely run opposite to the claim that the manipulation changed grades for the better up to 7 months after the intervention. The majority of students who received the growth mindset intervention demonstrated numeric declines in grade point averages from before to after the intervention. When examining post-intervention grades only, the majority of intervention students experienced a numeric increase in grades from semester 1 to semester 2, though a large proportion (roughly a quarter) of participants who received the intervention experienced a numeric decline in grades following the intervention. 

### 3.5. Treatment–Control Pair Comparisons of Grade Changes

We next compared changes in grades between treatment and control students. We first assessed treatment students’ change in grades from pre-intervention to post-intervention semester 1 relative to control students’ change in grades during the same time period. We calculated change scores for students, examining all possible treatment–control pairs. This assessment can provide a better comparison than within-subjects comparisons alone, as there could be a general trend of decreasing grades that the growth mindset intervention helps ameliorate. We then compared the change in grades from pre-intervention to post-intervention semester 2 and post-intervention semester 1 to post-intervention semester 2, examining all possible treatment–control pairs. As can be seen in Table 1, the results of the individual-level comparisons were near chance (i.e., 50%) as to whether the treatment student demonstrated greater numerical improvement relative to their control student counterpart. 

### 3.6. Treatment–Control Pair Comparisons of Post-Intervention Grades

We next assessed the evidence for the claim that students who received the intervention had better grades over freshman year than students in the control group. We examined all possible treatment–control student pairs with grades at post-intervention semester 1 and post-intervention semester 2 (the two time points in students’ freshman year), respectively. As can be seen in Table 1, the results were close to chance (i.e., 50%) as to whether the treatment student had numerically higher grades than their control student counterpart or whether the treatment student had numerically lower grades than their control student counterpart. 

### 3.7. Discussion

Though Yeager et al. (2016) implied that treatment students experienced a positive change in grades, they did not conduct any analyses examining grades changes. We, therefore, cannot compare the persons as effect sizes of grade change with effect sizes from aggregate analyses. Yeager et al. did, however, report that the intervention condition demonstrated higher GPAs at post-intervention semester 1 than the control group, *p* = 0.016, *d* = 0.279, and that the same intervention effect on GPA outcomes was observed in the following semester (post-intervention semester 2), *p* = 0.020, *d* = 0.269. These aggregate-level results support Yeager et al.’s claim that students who received the intervention had better grades over freshman year than students in the control group. However, by examining persons as effect sizes, we can observe that it was a nearly 50/50 chance that a given treatment student had numerically higher grades than a given control student.

The near-chance results suggest either that (a) the growth mindset intervention had little bearing on student grades (i.e., variability was mostly random), or (b) that the intervention was nearly equally likely to increase some students’ grades as it was to decrease other students’ grades (or some combination of the two). If variability was random and the intervention had little to no effect on student grades, this raises the question as to whether mindset interventions akin to that of Yeager et al. (2016) are worthwhile, considering the time and money spent. If the intervention is likely to decrease the grades of many students, teachers and policymakers must decide if raising the grades of some students is worth lowering the grades of others.

## 4. Ehrlinger et al. (2016)

### 4.1. Introduction

Ehrlinger et al. (2016) conducted a series of three studies examining the role of mindset on overconfidence and preferential attention. Ehrlinger et al. (2016) argued that both overconfidence and attention allocation are important avenues for academic research, as they can help elucidate ways to improve students’ learning trajectories. Students were assessed on overconfidence and attention allocation during the completion of practice problems on the GRE, a common standardized exam assessing aptitude and achievement outcomes for prospective graduate students. 

Ehrlinger et al. (2016) argued that overconfidence is most prevalent among students with fixed mindsets, because their mindset “leads them to forego learning opportunities in order to maintain positive beliefs regarding their competence” (p. 95). That is, they reported that students who view their intelligence as fixed (i.e., hold a fixed mindset) are likely to overestimate their performance because they focus on easy problems, which in turn may lead to reductions in learning. In contrast, they argued that students who view their intelligence as malleable (i.e., hold a growth mindset) have better self-insight because they are more willing to focus on difficult problems. Here, we focus on the claims associated with mindsets and attention allocation, as this is the presumed antecedent to learning and academic performance. 

In their first and third studies, Ehrlinger et al. examined the relationship between pre-existing student mindsets and overconfidence. These studies do not include a growth mindset manipulation and are not included here. In their second study, Ehrlinger et al. attempted to manipulate mindsets and measure group differences on attention allocation and overconfidence. We focus on Ehrlinger et al.’s (2016) study 2, where the study authors made claims about the effect of a mindset manipulation on attention allocation. Results on overconfidence can be found in the Supplemental Materials File S1.

In study 2, Ehrlinger et al. (2016) sought to experimentally manipulate mindset to test its effect on overconfidence, and whether differences in attention mediated the effect. Ninety-four university students were either assigned to read an article designed to teach students that intelligence is stable (fixed-mindset condition) or an article designed to teach students that intelligence is malleable (growth mindset condition). Attention allocation was determined by the number of seconds allocated to the easy problems and the difficult problems each relative to the overall time spent on the GRE practice problems. Ehrlinger et al. (2016) reported that “[t]eaching a growth mindset makes students open to difficulty” (p. 94). Ehrlinger et al. further explained that “[p]articipants who were randomly assigned to a condition in which they were taught an entity [i.e., fixed] (vs. incremental [i.e., growth]) view of intelligence subsequently allocated less time to difficult problems” (p. 98).

### 4.2. Methods

To assess the strength of the evidence that “[t]eaching a growth mindset makes students open to difficulty” (p. 94), we examined within-subjects comparisons by calculating the percentage of students taught a growth mindset who spent numerically more of their total time on difficult problems than on easy problems, the percentage who spent identical amounts of time on easy and difficult problems, and the percentage who spent numerically more of their total time on easy problems than on difficult problems.

We do not include a comparison of difference scores on difficult relative to easy attention allocation between students taught a growth mindset relative students taught a fixed mindset for two reasons. First, differing from growth mindset interventions where claims about improvements to student outcomes are often contextualized relative to a control group, this study did not include a control group. Thus, claims about the effect of a growth mindset manipulation on students should not be compared with a fixed-mindset condition unless specifically contextualized as such. Any results from a growth–fixed comparison cannot disentangle effects from a growth mindset treatment from effects from a fixed-mindset treatment. Second, Ehrlinger et al. provide their own between-subjects claim, which we assessed next.

To assess the strength of the evidence that “[p]articipants who were randomly assigned to a condition in which they were taught an entity [i.e., fixed] (vs. incremental [i.e., growth]) view of intelligence subsequently allocated less time to difficult problems” (p. 98), we examined each possible fixed-mindset condition–growth mindset condition pair and assessed the percentage of pairs in which the student in the fixed-mindset condition allocated a numerically smaller proportion of the total time spent to difficult problems compared with their growth mindset condition counterpart, the percentage of pairs who allocated an identical proportion of time to difficult problems, and the percentage of pairs in which the student in the fixed-mindset condition allocated a numerically greater proportion of the total time to difficult problems compared with their growth mindset condition counterpart. 

Two students in the growth mindset condition were missing values for the number of seconds taken on certain easy problems, thus impacting their reporting of total time that were allocated to easy problems. These students were excluded from the analyses.

### 4.3. Descriptive Results

Ehrlinger et al. (2016) reported that the overall time students spent on the test ranged from 159 to 929 seconds, with an average of 356.90 total seconds. Students spent between 7.60 and 80.00 seconds on the easy problems (*M* = 25.40 s), and between 9.60 and 48.40 s on the difficult problems (*M* = 21.19 s). 

Ehrlinger et al. (2016) reported that students in the fixed-mindset condition allocated less attention to difficult problems, *M* = 15.70 (*SE* = 1.026), than those in the growth mindset condition, *M* = 17.22 (*SE* = 1.023). Those in the fixed-mindset condition also allocated more attention to easy items, *M* = 20.94 (*SE* = 1.028), than those in the growth mindset condition, *M* = 18.54 (*SE* = 1.026). In both conditions, on average, students directed more time and attention toward easy problems than difficult problems.

### 4.4. Growth Mindset Condition Attention Allocation

Ehrlinger et al. (2016) claimed that “[t]eaching a growth mindset makes students open to difficulty” (p. 94). To assess the strength of the evidence that teaching a growth mindset makes students open to difficulty, we examined the amount of time students who received the growth mindset intervention spent on difficult, relative to easy, problems. As can be seen in Table 2, the results of this analysis indicate that the majority of students taught a growth mindset spent more time on the easy problems, contradicting the implication that these students would take more time to focus on the difficult problems than on the easy ones. 

Ehrlinger et al.’s aggregate results also do not support their claim that teaching a growth mindset makes students open to difficulty. Students in the growth mindset condition spent a similar amount of time on easy and difficult problems. The claim appears to be based on attention allocation relative to students who were taught a fixed mindset. Ehrlinger et al. observed a significant mindset manipulation × attention allocation interaction, with students in the fixed-mindset condition spending more time on easy compared with difficult problems (*p* < 0.001, η_p_^2^ = 0.32) than students taught a growth mindset (*p* < 0.10, η_p_^2^ = 0.04). Without a control group, we cannot know whether a growth mindset manipulation makes students (relatively more) open to difficulty or if a fixed-mindset manipulation makes students less open to difficulty.

### 4.5. Fixed vs. Growth Mindset Condition Pair Comparisons

To assess the claim that being taught a fixed mindset reduces attention to difficult problems compared with being taught a growth mindset, we examined how often students in the fixed-mindset condition spent numerically less of their total time on difficult problems compared with students in the growth mindset condition. As shown in Table 2, there is some evidence to support the claim that many of those in the fixed-mindset condition were more likely to allocate less attention to difficult problems than students in the growth mindset condition. Student behavior was in line with prediction for about two-thirds of the pairs and counter to prediction for about one-third of the pairs.

### 4.6. Discussion

Upon examination of the individual-level data, we reveal that the majority of participants taught a growth mindset spent more time on easy problems than on hard problems. However, when comparing students taught a fixed vs. a growth mindset, the majority of pairs, about two-thirds, behaved according to Ehrlinger et al.’s claim. Future researchers may wish to disentangle whether the growth mindset manipulation, the fixed-mindset manipulation, or both are driving the effect. Additionally, future researchers may wish to investigate Ehrlinger et al.’s suggestion that attention allocation to difficult problems is critical for student long-term learning. No evidence is reported in Ehrlinger et al. (2016) to support this suggestion; therefore, we cannot evaluate that claim here. Taken together, more evidence is needed to evaluate the importance of mindset on attention allocation.

## 5. Porter et al. (2022)

### 5.1. Introduction

Porter et al. (2022) recruited 50 sixth- and seventh-grade teachers who were randomly assigned to deliver the growth mindset intervention “Brainology” to their students or to serve in the control condition (total student *N* = 1996). Teachers and students in the control group were not given a task comparable to Brainology; the control classes were “teaching as usual”. Porter et al. (2022) described the intent of the mindset intervention as instilling growth mindset beliefs (i.e., that intellectual abilities can improve with learning and effort) in both teachers and students and fostering a supportive environment within the classroom. Therefore, the intervention had the ultimate goal of not only changing mindsets and improving academic outcomes for students, but to also change the beliefs of teachers who may hold a more fixed-oriented mindset. 

Porter et al.’s (2022, https://osf.io/z2nvy/ accessed on 20 July 2022) dataset has a number of limitations. First, they intended to recruit only science teachers; however, due to recruitment problems, they expanded to include a small number of math and English teachers, making some subgroup comparisons difficult. Second, each teacher designed their own curriculum using Brainology; therefore, the content, style, and duration varied by teacher. Third, Porter et al. have a large amount of missing data and/or non-comparable data. For example, some students’ grades were not consistently reported on the same scale and varied in their measurement precision. Finally, in some cases they did not have the data available to support their claims.

Porter et al. (2022, https://osf.io/z2nvy/ accessed on 20 July 2022) collected from the intervention and control groups measures of pre- and post-intervention teacher mindsets, pre- and post-intervention student mindsets, and pre- and post-intervention student grades for teachers and students in the treatment and control groups. Porter et al. (2022) report multiple positive effects from the intervention. For clarity, we delineate each major claim, our methods to assess the claim, and our results, before moving to the next claim.

### 5.2. Claim #1: Brainology Increases Growth Mindsets of Teachers and Students

Porter et al. (2022) report that “teachers’ growth mindsets increased as a result of delivering the intervention” (p. 1094) and that “we examined the effect of Brainology on student growth mindsets and found the predicted increases” (p. 1090).

#### 5.2.1. Methods

Porter et al. assessed teachers’ mindsets using a growth mindset questionnaire and then combined these scores with responses on a failure beliefs questionnaire, referring to this combined score as teachers’ mindsets. In contrast, Porter et al. only used the growth mindset questionnaire when assessing students’ mindsets. We used responses on the growth mindset questionnaire to assess teachers’ and students’ mindsets.

To assess the robustness of Porter et al.’s claims about the intervention increasing teacher and student growth mindsets, we calculated the number of teachers who administered the intervention who saw a numeric shift in their mindset toward a growth mindset from before to after the intervention. We also calculated the number of teachers who experienced no change in their mindset and the number of teachers administering the intervention who shifted toward more of a fixed mindset. We conducted these same analyses for the students who received the Brainology intervention. We also compared the mindset change scores from pre- to post-intervention between each possible treatment–control pair. We conducted this analysis for both the teachers and the students.

#### 5.2.2. Results and Discussion

##### Changes in Teachers’ Mindsets

A total of 25 of the 50 teachers were assigned to deliver Brainology in their classrooms. Mindset scores were missing for 4 of the 25 teachers. To examine the strength of the claim that teachers’ mindsets became more growth-oriented from delivering the intervention, we examined numerical change in mindset from pre-intervention to post-intervention of the teachers who delivered Brainology. As shown in Table 3, the largest share of teachers experienced no change in mindset, though almost as many shifted to more of a growth mindset.

We next compared teachers’ change in growth mindset between treatment and control teacher pairs. As can be seen in Table 3, nearly two-thirds of teacher treatment–control pairs demonstrated intervention changes according to expectation. The remaining third were either identical to controls or experienced less of a numerical shift toward a growth mindset than their control teacher counterpart.

We assessed why the within-subjects changes in mindset for the teachers delivering the Brainology growth mindset intervention yielded less impressive percentages than the between-subjects treatment–control change in mindset comparisons. This difference appears to be largely driven by the majority of control teachers demonstrating a *drop* in growth mindset beliefs from pre- to post-intervention. It is unclear why teachers assigned to the control condition would experience any change in mindset. Future researchers may wish to investigate whether this pattern replicates, and if so, what factor or factors are responsible for this pattern.

Despite Porter et al. stating that “teachers’ mindsets also changed as a result of delivering the program” (p. 1091), Porter et al. did not analyze change in teachers’ mindsets. Thus, we do not have aggregate results with which to compare our individual-level analyses. Rather, they report the difference scores between treatment and control teachers following the intervention, β = 0.70, 95% CI = [0.32, 1.11]. This comparison does not account for changes in teachers’ mindsets from baseline to post-intervention.

##### Changes in Students’ Mindsets

We next examined changes in students’ mindsets. In total, Porter et al. (2022, https://osf.io/z2nvy/ accessed on 20 July 2022), report data for 2433 students. Of these 2433 students, 1145 received the intervention. To examine the strength of the claim that students’ mindsets became more growth-oriented from receiving the intervention, we examined numerical change in mindset from pre-intervention to post-intervention among students receiving the Brainology growth mindset intervention. Mindset scores were missing from 15% of students who received the intervention. As shown in Table 3, of the 976 students who received the intervention and had mindset scores at both time points, just over half numerically shifted to more of a growth mindset. One in four students numerically shifted to more of a fixed mindset following the growth mindset intervention. The remainder experienced no numerical change in mindset.

We then compared students’ numerical change in growth mindset who received the Brainology growth mindset intervention to students’ numerical change in growth mindset who served as controls. As can be seen in Table 3, similar to the within-subjects findings, just over half of the pairs demonstrated that the treatment student numerically shifted to more of a growth mindset than their control student counterpart. In over a third of the pairs, the control students’ mindset numerically changed more toward a growth mindset than their treatment student counterpart. 

Despite Porter et al. having stated that they “examined the effect of Brainology on student growth mindsets and found the predicted increases” (p. 1091), Porter et al. did not examine increases in students’ mindsets. Thus, we do not have aggregate results with which to compare our individual-level effect sizes. Rather, they report the difference scores between treatment and control students following the intervention, β = 0.34, 95% CI = [0.26, 0.43]. This comparison does not account for increases in students’ mindsets from baseline to post-intervention.

### 5.3. Claim #2: Brainology Increases Student Grades

#### 5.3.1. Introduction

Porter et al. (2022) examined student achievement within the Brainology classrooms—the classes with the intervention—and their control class counterpart where the same subject was taught. Brainology classrooms were primarily science classes; however, due to recruitment issues, a few English and math classes were used. Porter et al. (2022) titled their paper “Growth-Mindset Intervention Delivered by Teachers Boosts Achievement in Early Adolescence” and claimed that “students who received the intervention had higher grades than control students at the end of the year in the Brainology class” (p. 1091).

Porter et al. also tested whether grades outside of Brainology classrooms improved for students who received the intervention compared with control-group students in those same subjects. If students’ grades are only impacted in Brainology classes, this suggests that the effect may be from teachers who deliver the intervention changing how they grade rather than from the growth mindset intervention influencing students’ beliefs. In contrast, if the intervention increased grades outside of Brainology classrooms, this suggests that the intervention influenced students’ learning and behavior. Porter et al. reported that “there was evidence that the intervention increased grades outside of Brainology classrooms” (p. 1093). 

We therefore assessed the claims that the intervention positively changed treatment students’ grades both within and outside of Brainology classrooms and that treatment students had higher post-intervention grades than control students. We used within-subjects comparisons, between-subjects comparisons of change scores, and post-intervention between-subjects comparisons. 

#### 5.3.2. Methods

We discovered multiple limitations with the Porter et al. dataset when conducting these calculations. First, 20% of the course grades for students were missing at pre-intervention and/or at post-intervention. Second, sometimes students’ pre-intervention grades appeared to be a numerical conversion from a letter grade, e.g., “3”, presumably because their course grade was a “B”, whereas some of the pre-intervention grades and all post-intervention grades were recorded on a scale of 0–100. We excluded students with these conversions from this analysis because a “3” could be a range of values on a 100-point scale.

The missing and/or non-comparable grade data were substantial. For science grades, 54% of students were either missing grades or were missing comparable pre- and post-intervention grades for their science classes. Likewise, for math grades, 54% of students were either missing grades or were missing comparable pre- and post-intervention grades. Similarly, for English grades, 53% of students were either missing grades or were missing comparable pre- and post-intervention grades.

We first assessed the robustness of the within-subjects claim that the intervention boosted achievement. We calculated how many students in the intervention experienced an increase in grades in their Brainology class from pre- to post-intervention, how many experienced no change in their grades, and how many experienced a decline in their grades. Students received the Brainology intervention either in their science class, their math class, or their English class.

We next assessed whether the intervention boosted grades from pre- to post-treatment when compared to control student outcomes. Using students with complete pre- and post-intervention numerical grades, we calculated the percent of all possible intervention-control pairs where the treatment student numerically improved their grade to a greater extent in their science Brainology course, shifted their grade to an identical extent, or had their grade numerically improve *less* than their control student counterpart. We repeated these calculations for students assigned to Brainology math classes and English classes, respectively, comparing change scores for pairs within the same course subject where the intervention or control had been administered.

We then assessed the strength of the between-subjects claim that students who received the intervention had higher grades at the end of the year in their Brainology course than control students in the same course. We calculated the persons as effect sizes for Brainology science classes, Brainology math classes, and Brainology English classes so that we were comparing grades in the same course subject.

Following persons as effect sizes calculations of the efficacy of the Brainology intervention on student performance in intervention courses, we then calculated effect sizes for the claim that the intervention had beneficial spillover effects for classes outside of the Brainology classroom. We examined the evidence for the within-subjects claim that the intervention boosted grades outside of Brainology classrooms. We calculated how many students who received a Brainology intervention in one subject experienced an increase in grades in classes outside the intervention pre- to post-intervention, how many experienced no change, and how many experienced a decline in grades pre- to post-intervention. 

We next examined whether the intervention boosted grades for courses outside of Brainology from pre- to post-intervention when compared with grade changes of control student counterparts. Again, we conducted these analyses for each of the Brainology class subjects. Finally, we conducted between-subjects analyses examining the end-of-year grades for all possible pairs of intervention and control students for classes outside the Brainology class to assess potential spillover effects with a post-intervention between-subjects approach.

#### 5.3.3. Results and Discussion

To assess the strength of the evidence for the claim that the intervention boosted achievement, we examined the pre- to post-intervention changes in grades for the 493 students who were in the intervention condition and who had complete and comparable grade data at pre- and post-intervention. We first examined changes in grades in their Brainology class (the class in which they received the intervention). As shown in Table 4, depending on the course, between one-third and two-thirds of the students experienced improved pre- to post-intervention grades, and between a quarter and over half of students experienced a decline in grades from pre- to post-intervention.

Next, we assessed whether the intervention boosted grades by comparing change scores of pairs of intervention and control students. The pattern was largely similar to the within-subjects results. As shown in Table 4, around half of treatment students showed greater numerical grade improvements from pre- to post-intervention than their control student pairs. Between a third and half of treatment students had less positive numerical change than their control-group counterpart, depending on the course. Despite Porter et al. having stated that the intervention boosted achievement, Porter et al. did not examine changes in students’ grades. Thus, we do not have aggregate results with which to compare our individual-level effect size results.

To assess the strength of the evidence for the claim that students receiving the intervention had higher grades in the class in which they received the intervention at the end of the year than the control group, we examined each treatment–control student pair. We first considered Brainology classes (i.e., classes where the treatment students received the intervention) and compared grades with their control group counterparts in the same subject. As shown in Table 4, in around half to two-thirds of the pairs, the student who received the intervention outperformed their control student counterpart, and in around a third to nearly half of the pairs, the student in the control group outperformed their treatment student counterpart. Porter et al. report that students in the intervention group had higher grades than the control group in their Brainology class by around 2.40 grade percentage points, β = 0.23, 95% CI = [0.07, 0.39]. This aggregate effect does not capture the pattern of individual-level results. For example, in science, the largest group, for almost every student receiving the intervention who experienced numerically better grades than their control group counterpart, there was a student who experienced numerically worse grades than their control group counterpart. The results for math were similar to those for science, whereas the results for English (the smallest group) were slightly more in favor of the intervention and more in line with the aggregate results.

Following the calculation of the effect sizes for Brainology classroom performance, we examined academic performance outcomes outside Brainology classrooms (e.g., students’ math class grades if they received the intervention in their science class). As shown in Table 5, these results suggest that the intervention improved some students’ grades while worsening other students’ grades. With the exception of English grades for students who received the math class Brainology intervention, the minority of students’ grades numerically improved. Porter et al. stated that “there was evidence that the intervention increased grades outside of Brainology classrooms” (p. 1093). However, Porter et al. did not examine change scores. Thus, we cannot compare our individual-level effect sizes to their aggregate effect sizes. 

We then compared the change scores of every pair of treatment–control students in the classes outside of the Brainology intervention. Some percentages are similar to the within subject results, whereas other percentages provide more evidence for Porter et al.’s claims. There is no discernable pattern. As shown in Table 5, there was a wide spread of change score comparison outcomes; proportions ranged from less than half to over two-thirds of pairs in which the treatment student showed greater numerical improvement in their grades relative to their control student counterpart. Conversely, sizeable percentages of treatment students experienced *less* improvement than their control student counterpart, with between a quarter to nearly half of pairs performing counter to expectation.

We next examined between-subjects comparisons of post-intervention grades, which was analogous to Porter et al.’s aggregate analyses. As shown in Table 5, when students received the intervention in their science class, around half of treatment students numerically outperformed their control-student counterpart, and in around half of the other pairings, the control student numerically outperformed the treatment student. Only when students received the Brainology intervention in their English class were individual-level analyses supportive of a spillover effect. Oddly, Porter et al. claimed that “there was evidence that the intervention increased grades outside of Brainology classrooms” (p. 1093) even though their own aggregate analyses yielded null results: β = 0.14, 95% CI = [−0.02, 0.30].

### 5.4. Claim #3: Brainology Increases Grades of Lower-Achieving Students

#### 5.4.1. Introduction

Porter et al. (2022) examined heterogeneity of effects and reported that “Brainology improved the grades of lower achieving students in the target class” (p. 1093). Porter et al. used the entire range of pre-intervention grades and computed marginal tests at ± one standard deviation. They considered students as lower achieving whose grades were one standard deviation or lower than the mean pre-intervention.

#### 5.4.2. Methods

We considered students with a pre-intervention grade one standard deviation or lower than the mean as being lower-achieving. To assess the robustness of the within-subjects claim that the intervention increased grades of lower-achieving students in the target (i.e., Brainology) classes, we calculated within-subjects persons as effect sizes. We used grades that were on the same scale as, and therefore comparable to, post-intervention grades. We first calculated the number of lower-achieving students who received the intervention and experienced a numerical increase in grades (pre- to post-intervention) in their Brainology class, the number who experienced no numerical change, and the number who experienced a numerical decline in grades in their Brainology class. We examined grade changes within science and math courses only because only three lower-achieving students received the intervention in their English class.

As an extension of assessing the claim of whether the intervention improved the grades of lower-achieving students in their targeted class, we also compared the change scores from pre- to post-intervention between lower-achieving students in the intervention and their control student counterparts. We examined grade changes within science and math courses only, because there were not enough lower-achieving students who received the intervention in the English class for meaningful comparisons. 

We also compared end-of-year grades between lower-achieving students in the treatment and control conditions. Again, we only examined science and math courses because there were not enough lower-achieving students in the English classes for meaningful comparisons.

#### 5.4.3. Results and Discussion

Porter et al. (2022) claimed that intervention effects on grades were strongest for lower-achieving students after observing a significant intervention × pre-intervention grades interaction: β = −0.12, 95% CI = [−0.20, −0.04], where larger effects on grades were observed for lower-achieving students (β = 0.27, 95% CI = [0.10, 0.44]) than higher-achieving students (β = 0.07, [−0.10, 0.25]). 

The within-subjects persons as effect sizes demonstrated that a large majority of the lower-achieving students who received the intervention did in fact have numerically improved grades from pre- to post-intervention in their Brainology classes, at least in science and math (there were not enough relevant students in English to meaningfully evaluate), see Table 6. 

However, many of the lower-achieving students in the control group also demonstrated improved grades. Table 6 shows the persons as effect sizes for pre- to post-intervention grade change differences between treatment and control student pairs. Nearly two-thirds of lower-achieving students in the science Brainology intervention showed greater numerical improvement to their academic performance in the course than their control group counterpart. However, when examining change scores for math grades between groups, over half of the control students in treatment–control pairs demonstrated a greater numerical improvement in grades than their treatment student counterpart. Again, Porter et al. did not analyze change scores in their study; therefore, we cannot compare our results directly to theirs. 

Table 6 also shows that, when examining post-intervention grades in Brainology science and math classes, the student receiving the intervention had a numerically higher grade than their control student counterpart just over half the time in science and less than a third of the time in math. Taken together, there is not a consistent pattern of persons as effect sizes that strongly supports Porter et al.’s claim that the intervention improved lower-achieving students’ grades.

### 5.5. Claim #4: Brainology Is Most Effective When Teachers Initially Have Fixed Mindsets

#### 5.5.1. Introduction

Notably, Porter et al. (2022) report that students who underwent the intervention saw the greatest improvements in grades when taught by teachers with pre-intervention fixed mindsets. They stated that “[t]he effects were largest for students whose teachers endorsed fixed mindsets before the intervention” (p. 1086). Their explanation for this finding was that students in classrooms with teachers who endorsed fixed mindsets would have the most to gain from the changing classroom context. 

Porter et al. determined teachers’ mindsets based on two questionnaires on a 1–5 scale, assessing both mindset and failure beliefs. These responses were averaged to create an individual’s composite score, where responses > 3 indicated disagreement with fixed-mindset statements, and responses < 3 indicated more agreement with fixed-mindset statements. In contrast, Porter et al. only used the mindset scale when assessing students’ mindsets.

We clarify here that Porter et al. conducted their analyses by treating mindset as a continuous variable. However, when interpreting their results, Porter et al. referred to teachers as having a fixed mindset or having a growth mindset. Additionally, rather than referring to teachers who agreed more with fixed-mindset statements as having a fixed mindset, they referred to teachers who disagreed with mindset statements—but less strongly than other teachers—as having a fixed mindset. That is, they treated “less of a growth mindset” as synonymous with “holding a fixed mindset”. This definition is only explained in a footnote. We sought to explore the effects of the intervention on students who had a teacher endorse fixed-mindset statements as their claim suggests.

#### 5.5.2. Methods

Unlike Porter et al. (2022), we examined teacher’s mindsets based on their responses to the mindset questionnaire rather than by combining these responses with failure beliefs. Porter et al. measured mindset with four items, reverse-coded, on a 1–5 scale, where responding to a statement with a ‘5’ indicated strong disagreement with a fixed-mindset statement (after reverse coding) and responding with a ‘1’ indicated strong agreement with a fixed-mindset statement (after reverse coding). Thus, an average response greater than 3 indicated that teachers generally disagreed with fixed-mindset statements. In contrast, an average response of less than 3 indicated had teachers generally endorsed fixed-mindset statements. 

Additionally, differing from Porter et al., we did not define teachers who mostly disagreed with fixed-mindset statements as having a fixed mindset. Instead, we defined teachers as having a fixed mindset if they generally agreed with fixed-mindset statements. Only one teacher in Porter et al.’s (2022, https://osf.io/z2nvy/ accessed on 20 July 2022) dataset endorsed a fixed mindset before the intervention. This teacher’s average response before the intervention was 2.75 on the 1–5 scale, whereas all other teachers scored ≥ 3.25 on this scale, which indicated general disagreement with a fixed mindset.

The sole teacher with a pre-intervention fixed mindset administered the intervention in their science classroom. To assess the claim that intervention effects were largest for students whose teachers initially endorsed fixed mindsets, we examined how many students in the classroom of the teacher with a pre-intervention fixed mindset saw an increase in grades, how many saw no increase, and how many saw a decline in grades. We could not compare intervention and control grades of students whose teachers endorsed fixed mindsets because no teacher with a fixed mindset served as a control teacher. 

#### 5.5.3. Results and Discussion

Twenty-five students received the Brainology intervention from the sole teacher with a pre-intervention fixed mindset in the study, and there were no teachers in the control condition endorsing fixed mindsets with which comparisons to the intervention could be made among their students. In total, 5 of these students’ post-intervention grades were missing, leaving 20 students to examine. As shown in Table 7, in their science Brainology class, only a quarter of these students’ grades numerically improved from pre- to post-intervention, nearly half experienced no numerical change, and over a quarter of students’ grades numerically worsened. 

When examining effects in non-Brainology classes, a minority of students experienced numerically improved grades from pre- to post-intervention; the remainder experienced no change or a numerical decline in grades from pre- to post-intervention. Because of the limited nature and size of this sample, our analysis of the 20 students with a single teacher holding a fixed mindset is not very informative, and we cannot draw robust conclusions about the effectiveness of the intervention. Thus, these results cannot substantiate the claim that Brainology is most effective for students whose teachers initially have a fixed mindset.

Porter et al. found a significant intervention × teacher pre-intervention mindset effect on student grades, β = −0.47, 95% CI = [−0.82, −0.13], where students in the intervention with teachers disagreeing with fixed-mindset statements, but less strongly disagreeing than other teachers, had higher post-intervention grades (4.16 percentage points higher) relative to students in the control group who had teachers who less strongly disagreed with fixed-mindset statements, β = 0.40, 95% CI = [0.20, 0.60]. In contrast, students with teachers who strongly disagreed with fixed-mindset statements did not demonstrate a significant intervention effect, β = −0.08, 95% CI = [−0.29, 0.13]. Porter et al. interpreted these results as though treatment students with teachers *with fixed mindsets* had higher post-intervention grades than control students with teachers with fixed mindsets. With one teacher endorsing a fixed mindset, Porter et al. did not have the data to make claims about teachers who endorsed fixed mindsets. At most, they could have made claims about teachers who disagreed with fixed mindset statements but less strongly relative to others in the sample.

### 5.6. Claim #5: Brainology Is Most Effective for Lower-Achieving Students Whose Teachers Initially Have Fixed Mindsets

#### 5.6.1. Introduction

Porter et al. claimed that the intervention was most effective for lower-achieving students whose teachers had fixed mindsets pre-intervention because the intervention changed how teachers graded. They stated, “Given that the greatest impact was found for students whose teachers initially had fixed mindsets, it is possible that the program reduced bias in grading. Rather than assigning grades on the basis of low prior expectations, teachers may have more accurately perceived the academic growth of students who were initially lower achieving” (p. 1094). Porter et al. continued: “It is notable that Brainology worked primarily for teachers who started the study with fixed mindsets. This finding suggests a compensatory effect wherein the intervention made up for the negative toll that having a teacher with a fixed mindset typically takes on lower achieving students’ grades” (p. 1094).

Again, we note here that Porter et al. (2022) defined teachers who disagreed with fixed-mindset statements, but less strongly relative to others in the sample, as having fixed-mindset beliefs. We aimed in our persons as effect sizes to look at intervention effects for students who had a teacher who initially endorsed fixed-mindset beliefs according to Porter et al.’s stated claims.

#### 5.6.2. Methods

To assess the claim that Brainology is most effective for lower-achieving students whose teachers initially had fixed mindsets, we examined how many lower-achieving students in the one classroom where a teacher had a fixed mindset experienced numerically improved grades, no change in grades, and a numerical decline in grades.

#### 5.6.3. Results and Discussion

Zero students were lower-achieving in the classroom of the one teacher who initially held a fixed mindset. In their aggregate analysis, Porter et al. (2022) claimed that having characteristics of low pre-intervention student performance, coupled with having a teacher with pre-intervention fixed-mindset beliefs, led to the greatest benefits. Porter et al. did not have data on teachers with fixed mindsets, as only a single teacher initially held a fixed mindset, and they did not have data on lower-achieving students who had a teacher initially holding a fixed mindset.

### 5.7. Porter et al. (2022) Discussion

Porter et al. (2022) made five major claims. Examining persons as effect sizes indicated that many students and teachers behaved or performed inconsistently with Porter et al.’s claims. The exception to this is for lower-achieving students’ change in grades, where the majority improved following the intervention. However, when comparing lower-achieving students who received the intervention to those who did not, the results were once again unimpressive; the lower-achieving students in the control group frequently performed as well or better than the lower-achieving students who received the intervention.

Furthermore, Porter et al. made several claims where they did not have the data to make such a claim. Notably, Porter et al. described teachers who disagreed with fixed mindset beliefs as holding a fixed mindset. They claimed that the intervention was most effective when students’ teachers initially held a fixed mindset, despite only a single teacher in the sample initially holding a fixed mindset. They further claimed that lower-achieving students from these classrooms demonstrated large effects, despite there being no lower-achieving students in the single classroom where the teacher initially held a fixed mindset. 

## 6. General Discussion

Our study of three mindset interventions is a step toward answering the call for a heterogeneity revolution. We considered the full range of treatment effect heterogeneity at the student level, from benefits to detriments. Examining the complete range at this level allows readers to better understand heterogeneity of outcomes.

We used a recently developed individual-level effect size. The persons as effect sizes method developed by Grice et al. (2020) is designed to calculate the percentage of participants who behaved or responded according to theory and the percentage who did not. We extended this method by differentiating the percentage of participants who demonstrated no numerical change/difference in response or performance from the percentage of participants who behaved or responded counter to expectations. Exploring the whole range of outcomes at the individual level is often obscured in aggregate analyses; yet, it is important for understanding variation in outcomes and making well-informed policy decisions.

We examined three papers that claimed, based on aggregate analyses, that growth mindset interventions can improve academic performance of students. Using aggregate analyses, Yeager et al. (2016) claimed that the intervention improved students’ grades. Examining individual-level data, we found that the majority of students who received the intervention conducted by Yeager et al. (2016, https://osf.io/fm5c2/ accessed on 15 July 2022) experienced a decline in grades from pre- to post-intervention. When comparing treatment and control participants, we found that nearly half the pairs showed that the treatment student performed worse than their control student counterpart. These results suggest that either change in grades is largely due to chance, or that the intervention is about as likely to benefit some students as it is to harm other students.

Likewise, using aggregate analyses, Ehrlinger et al. (2016) claimed that students taught a growth mindset spend more time solving difficult problems. Examining individual-level data, we found that nearly two-thirds of students taught a growth mindset spent less time on difficult problems than on easy problems. When comparing growth-mindset and fixed-mindset condition participants, we found that about two-thirds of the growth-mindset condition students focused more on difficult problems than their fixed-mindset condition counterpart, and about one-third of students taught a growth mindset spent more time on the easy problems than their fixed-mindset condition counterpart. These results suggest that teaching a growth mindset may influence some students to shift their focus more to difficult problems, though it may be that teaching a growth mindset has no impact but that teaching a fixed mindset is what influences them to focus on easy problems.

Porter et al. (2022) made five major claims. In most cases, individual-level effect sizes generally did not strongly corroborate their conclusions. For example, their aggregate analyses led them to claim that administering the intervention shifted teachers’ mindsets toward growth. However, the largest share of teachers had identical mindsets before and after the intervention. Likewise, Porter et al. claimed that undergoing the intervention shifted students’ mindsets toward growth. However, nearly half the students reported no change or reported less of a growth mindset following the growth mindset intervention. In addition, when comparing treatment–control student pairs in Porter et al. (2022, https://osf.io/z2nvy/ accessed on 20 July 2022), in most of the classes, around half the time the treatment student performed worse than their control student counterpart. These results suggest that either results are due to chance, or that the intervention benefits some students while harming others.

For one claim, some of the individual-level effect sizes supported the aggregate analyses. We found that a large majority of lower-achieving students, defined as students with pre-intervention grades one standard deviation or lower than the mean, improved from pre- to post-intervention. However, many lower-achieving students in the control group also saw an improvement. When comparing changes in grades between the two groups, the effect sizes no longer strongly supported Porter et al.’s conclusions. 

Finally, some of Porter et al.’s claims were made based on treating teachers disagreeing with fixed mindset statements, but not strongly, as being synonymous with agreeing with fixed-mindset statements. The most notable claim made by Porter et al. (2022) was that the student intervention was most impactful when teachers initially held a fixed mindset. There was only one teacher who initially agreed with fixed-mindset statements. Within this teacher’s classroom, only a quarter of the students improved their grades from pre- to post-intervention. Furthermore, Porter et al. claimed that lower-achieving students benefited most from the intervention when their teachers initially held fixed mindsets, but there were zero lower-achieving students as defined by Porter et al. in the class of the sole teacher who initially endorsed a fixed mindset.

Across the evaluations of the claims in these three papers, we have shown that when individual-level effect sizes are considered, the evidence is not as strong as is implied when one examines aggregate results alone, even when examining sub-samples hypothesized to see the most benefit. When considering students and teachers with disparate backgrounds, beliefs, and needs, our effect sizes allow us to conclude that some individuals who are promised to see the greatest improvements may, in fact, experience no positive outcomes or even detriments.

### Limitations and Future Directions

Our study of persons as effect sizes within the growth mindset intervention literature is not a comprehensive or systematic review. Rather, our study was based on published literature with preexisting and available open data. We only included published studies to evaluate public claims. We made this decision because the claims in unpublished manuscripts may evolve prior to publication, and unpublished datasets without an accompanying manuscript would not have claims for us to evaluate. 

Our primary barrier encountered to including more studies was the literature itself. Of the relevant studies, few shared open data at the individual level. Though we searched for other relevant studies with open individual-level data, there may be studies that we missed. We know of no other studies that met our inclusion criteria other than these three studies. Future researchers may want to contact researchers to ask for data to analyze that was not publicly posted. Only including studies with posted open individual-level data may not be representative of the wider literature. 

There are multiple levels of heterogeneity one can examine. The present study primarily focused upon heterogeneity of individual responses (i.e., mindset and academic outcomes) from the intervention. However, some occupational researchers, educational researchers, and psychologists (i.e., Burnette et al. 2022; Domitrovich et al. 2008; Klein and Sorra 1996; Macnamara and Burgoyne 2022) have advocated for considering the quality of the intervention itself and/or the strength of the support system surrounding the intervention. The studies that we examined did not provide sufficient information about the support system at the school or macro levels for us to evaluate. We present this as an avenue for examination by researchers in the future, to explore heterogeneity at all levels of the implementation of mindset interventions (macro, school, and individual levels). 

The three studies reported here used disparate methods, samples, and approaches. We recommend that researchers attempt to replicate these and other studies. Bryan et al. (2021) suggest that attempted replications fail because the replication does not take into account heterogeneity. Replication and heterogeneity need not be at odds with one another. Rather, researchers should attempt to replicate studies and examine heterogeneity systematically.

Currently, intervention implementation and subgroups often vary from study to study, or, in the case of Porter et al. (2022), intervention implementation varied from teacher to teacher in the same study. With little consistency in domain, groups, method of delivery, or setting, it is difficult to establish replicable effects and difficult to examine heterogeneity systematically. In the present paper alone, the three studies differed in mindset domain (intelligence, personality), age group (ninth grade, university, or sixth and seventh grade), and outcomes (GPA, attention allocation). In addition, methods of delivery and settings for the intervention differed across these three studies: two 25-min interventions delivered to classrooms; an article intervention delivered in the laboratory; and an intervention delivered by teachers within a course subject. As it stands, understanding the underlying mechanisms and the conditions under which effects appear is hampered when methods and outcomes of mindset interventions vary from study to study. Researchers should strive to replicate effects under specific conditions to better understand heterogeneity of effects.

Finally, we recommend that researchers report individual-level effect sizes along with aggregate analytical results. The persons as effect sizes approach is not a replacement for inferential statistics and cannot test for statistical significance. Rather, this approach provides an effect size based on individual-level responses to consider how many participants are responding as expected alongside other statistics. Aggregate analyses, aggregate effect sizes, and individual-level effect sizes can be reported together to provide an additional layer of evidence for researchers and readers to evaluate. 

## 7. Conclusions

We considered heterogeneity in two ways. First, we analyzed three growth mindset interventions at the individual level. How does this increase our understanding of heterogeneity? Aggregating across students or subsamples of students leads to information loss and can obfuscate variance in responses to treatments. We used a straightforward, easy-to-understand approach to reveal how many students in each study or subgroup responded to the intervention in line with the study authors’ claims, and how many did not.

Second, we considered the full range of individual-level heterogeneity. We examined how many students responded positively (i.e., according to claims), how many appeared unaffected (i.e., not according to claims), and how many responded negatively (i.e., counter to claims). Understanding the full spectrum of effects informs educators’ decisions when weighing the costs, benefits, and risks of implementing interventions.

Our analyses suggest that growth mindset interventions might benefit some students while harming others. Academic performance decrements must be acknowledged and investigated to better understand the heterogeneity of growth mindset effects on academic outcomes. Interventions that improve the outcomes of some students—at the expense of others—are not ideal solutions, especially when considering disparities in academic outcomes.

We propose a call for a transparent and complete view of heterogeneity. First, we encourage mindset researchers in the future to openly share their data at the individual level. Second, we urge researchers to consider examining heterogeneity in multiple ways, and, along with aggregate statistical tests and aggregate effect sizes, to include the percentage of participants who behaved according to theory and those who behaved counter to theory. Providing both aggregate and individual effect sizes allows readers to contextualize the heterogeneity of the effects that mindset interventions can elicit—the beneficial effects, the lack of effects, and the detrimental effects—as well as whether these person-to-person effects are consistent with claims from aggregate analyses. Only by fully addressing heterogeneity can we deepen our theoretical understanding and improve interventions.

## Figures and Tables

**Table 1:** Students whose grades suggest a positive impact from the intervention (according to claim), no difference, and negative impacts (counter to claim) in Yeager et al.’s (2016) study 2.

Analysis	According to Claim	No Difference	Counter to Claim	*n*
Treatment students’ change in grades				
Pre- to post-intervention semester 1	29.61%	00.66%	69.74%	152
Pre- to post-intervention semester 2	37.50%	00.00%	62.50%	152
Post-intervention semester 1 to 2	60.26%	13.25%	26.49%	151
Treatment–control pair comparisons of grade change				
Pre- to post-intervention semester 1	56.79%	00.02%	43.19%	23,256
Pre- to post-intervention semester 2	57.81%	00.00%	42.19%	23,408
Post-intervention semester 1 to 2	52.59%	06.18%	41.23%	23,103
Treatment–control pair comparisons of post-intervention grades				
Treatment–control pairs at semester 1	52.23%	01.70%	46.07%	23,256
Treatment–control pairs at semester 2	53.68%	01.83%	44.49%	23,408

Note. *n* = number of treatment students or all possible treatment–control pairs. Treatment students’ change in grades = Percent of treatment students whose grades numerically increased, were identical, or decreased. Treatment–control pair grade change = Percent of all possible intervention-control pairs where the treatment student’s grades displayed a greater increase/lower decrease, the same change, or less of an increase/more or a decrease relative to their control student counterpart. Treatment–control pair comparisons of post-intervention grades = Percent of all possible treatment–control pairs of students where the treatment student had numerically higher grades, identical grades, or lower grades than their control student counterpart at that time point.

**Table 2:** Students who behaved in line with Ehrlinger et al.’s (2016) study 2 claims, showed no difference, or behaved opposite to predictions.

Analysis	According to Claim	No Difference	Counter to Claim	*n*
Students in growth mindset condition				
More time spent on hard than easy	38.78%	00.00%	61.22%	49
problems				
Fixed vs. growth mindset condition pair comparisons				
Student taught a fixed mindset				
spent *less* time on hard problems	68.63%	00.00%	31.37%	2107

Note. Ehrlinger et al. attempted to manipulate mindsets in this study. *n* = number of treatment students or all possible treatment–control pairs. According to claim = percentage of students taught a growth mindset who spent more time on hard than easy problems and percentage of pairs where the student taught a fixed mindset spent less time on hard problems than their growth mindset condition counterpart. No difference = percentage of students taught a growth mindset who spent identical amounts of time on hard and easy problems and percentage of pairs where the student taught a fixed mindset spent an identical amount of time on hard problems as their growth mindset condition counterpart. Counter to claim = percentage of students taught a growth mindset who spent less time on hard than easy problems and percentage of pairs where the student taught a fixed mindset spent more time on hard problems than their growth mindset condition counterpart.

**Table 3:** Teachers’ and students’ shift toward a growth mindset, lack of mindset change, and shift toward a fixed mindset according to Porter et al. (2022, https://osf.io/z2nvy/ accessed on 20 July 2022).

Analysis	According to Claim	No Difference	Counter to Claim	*n*
Teachers’ change in mindset				
Brainology intervention	42.86%	47.62%	09.52%	21
Treatment–control pair comparison	65.42%	19.47%	15.11%	483
Students’ change in mindset				
Brainology intervention	55.53%	18.75%	25.72%	976
Treatment–control pair comparison	53.48%	9.87%	36.65%	1,075,552

Note. *n* = number of treatment students or all possible treatment–control pairs. According to claim = percentage of teachers or students who shifted to more of a growth mindset from pre- to post-intervention or percentage of all treatment–control pairs where the treatment teacher or student shifted more toward a growth mindset than their control counterpart. No difference = percentage of teachers or students with identical pre- and post-intervention mindset mean scores or percentage of all treatment–control pairs where the treatment teacher or student had identical growth mindset change scores as their control counterpart. Counter to claim = percentage of teachers or students who shifted to less of a growth mindset from pre- to post-intervention or percentage of all treatment–control pairs where the treatment teacher or student shifted less toward a growth mindset than their control counterpart.

**Table 4:** Treatment students whose Brainology grades improved or were better than control students (according to claim), did not differ from baseline/control (no difference), or declined or were worse than control students (counter to claim) in Porter et al. (2022).

Analysis	According to Claim	No Difference	Counter to Claim	*n*
Brainology students’ grade changes (pre- to post-intervention) in their respective Brainology classes				
Science grades	59.94%	11.60%	28.45%	362
Math grades	67.39%	04.35%	28.26%	92
English grades	33.33%	07.69%	58.97%	39
Treatment–control pair grade change comparison (pre- to post-intervention)				
Science grades	57.43%	03.91%	38.66%	183,172
Math grades	61.32%	04.62%	34.06%	5980
English grades	43.77%	04.31%	51.92%	2184
Treatment–control pair post-intervention grade comparison				
Science grades	51.67%	03.53%	44.80%	510,340
Math grades	53.25%	02.88%	43.87%	28,674
English grades	66.99 %	01.99%	31.02%	21,684

Note. Brainology classes refer to the class where students received the intervention. *n* = number of treatment students or all possible treatment–control pairs. According to claim = percentage of treatment students whose grade numerically increased from pre- to post-intervention or percentage of all treatment–control pairs where the treatment student improved more or had a higher post-intervention grade than their control counterpart. No difference = percentage of treatment students with identical pre- and post-intervention grades or percentage of all treatment–control pairs where the treatment student had identical grade change scores or post-intervention grades as their control counterpart. Counter to claim = percentage of treatment students whose grade numerically decreased from pre- to post-intervention or percentage of all treatment–control pairs where the treatment student showed less improvement or a lower post-intervention grade relative to their control counterpart.

**Table 5:** Students whose non-Brainology grades improved or were better than control students (according to claim), did not differ from baseline/control (no difference), or declined or were worse than control students (counter to claim) in Porter et al. (2022).

Analysis	According to Claim	No Difference	Counter to Claim	*n*
Change in non-Brainology class grades for treatment students				
Brainology in science class				
Math grades	42.42%	15.15%	42.42%	363
English grades	49.45%	11.81%	38.74%	364
Brainology in math class				
Science grades	39.13%	06.52%	54.35%	92
English grades	51.09%	08.70%	40.22%	92
Brainology in English class				
Science grades	46.15%	15.38%	38.46%	39
Math grades	43.59%	17.95%	38.46%	39
Treatment–control non-Brainology class grade change comparisons				
Brainology in science				
Math grades	57.46%	04.20%	38.34%	187,308
English grades	47.85%	04.44%	47.71%	188,552
Brainology in math				
Science grades	57.66%	02.89%	39.45%	5980
English grades	69.67%	04.34%	25.99%	5980
Brainology in English				
Science grades	51.56%	03.93%	44.51%	2184
Math grades	66.21%	05.08%	28.71%	2184
Treatment–control non-Brainology class post-intervention grade comparisons				
Brainology in science				
Math grades	46.95%	03.06%	49.99%	516,306
English grades	47.01%	03.88%	49.11%	516,306
Brainology in math				
Science grades	50.64%	03.25%	46.11%	28,674
English grades	58.31%	03.15%	38.54%	28,674
Brainology in English				
Science grades	67.27%	02.54%	30.19%	21,528
Math grades	74.99%	01.84%	23.17%	21,528

Note. Non-Brainology classes refer to the classes outside of which students received either the intervention or control. *n* = number of treatment students or all possible treatment–control pairs. According to claim = percentage of treatment students whose grade numerically increased from pre- to post-intervention or percentage of all treatment–control pairs where the treatment student improved more or had a high post-intervention grade than their control counterpart. No difference = percentage of treatment students with identical pre- and post-intervention grades or percentage of all treatment–control pairs where the treatment student had identical grade change scores or had identical post-intervention grades as their control counterpart. Counter to claim = percentage of treatment students whose grade numerically decreased from pre- to post-intervention or percentage of all treatment–control pairs where the treatment student showed less improvement or a lower post-intervention grade relative to their control counterpart.

**Table 6:** Lower-achieving students whose grades improved or were higher than controls (according to claim), did not differ from baseline/control (no difference), or decreased or were lower than control (counter to claim) in Porter et al. (2022).

Analysis	According to Claim	No Difference	Counter to Claim	*n*
Brainology students’ change in grades				
Science grades	80.00%	12.73%	07.27%	55
Math grades	82.35%	05.88%	11.76%	17
Treatment–control change in grades comparison				
Science grades	63.62%	03.82%	32.56%	5940
Math grades	42.16%	06.86%	50.98%	204
Treatment–control pair post-intervention grade comparison				
Science grades	58.05%	07.27%	37.63%	5940
Math grades	31.86%	04.90%	63.24%	204

Note. Calculations were not conducted for English classes due to the small sample size. *n* = number of treatment students or all possible treatment–control pairs. According to claim = percentage of lower-achieving treatment students whose grade numerically increased from pre- to post-intervention or percentage of all lower-achieving treatment–control pairs where the treatment student improved more or had a higher post-intervention grade than their control counterpart. No difference = percentage of lower-achieving treatment students with identical pre- and post-intervention grades or percentage of all treatment–control pairs where the treatment student had identical grade change scores or post-intervention grades as their control counterpart. Counter to claim = percentage of lower-achieving treatment students whose grade numerically decreased from pre- to post-intervention or percentage of all treatment–control pairs where the treatment student showed less improvement or a lower post-intervention grade relative to their control counterpart.

**Table 7:** Students who received the intervention from a teacher who initially endorsed a fixed mindset whose grades improved (according to claim), remained the same (no difference), or worsened from pre- to post-intervention (counter to claim) in Porter et al. (2022).

Analysis	According to Claim	No Difference	Counter to Claim	*n*
Brainology class				
Science grades	25.00%	45.00%	30.00%	20
Non-Brainology classes				
Math grades	40.00%	35.00%	25.00%	20
English grades	45.00%	40.00%	15.00%	20

Note. Only one teacher in the sample held a pre-intervention fixed mindset. This teacher taught Brainology in a science class. Brainology class refers to the class where students received the intervention. Non-Brainology classes refer to classes students took other than the class in which they received the Brainology intervention. *n* = number of treatment students from the teacher with a fixed mindset.

## Data Availability

No new data were created in this study. The analyses presented in this study are from data openly available at https://osf.io/ufamv/ accessed on 15 July 2022 (Yeager et al. 2016), https://osf.io/fm5c2/ accessed on 15 July 2022 (Ehrlinger et al. 2016), and https://osf.io/z2nvy/ accessed on 20 July 2022 (Porter et al. 2022).

## Supplementary Materials

The following supporting information can be downloaded at: https://www.mdpi.com/article/10.3390/jintelligence11060104/s1, File S1: Ehrlinger et al. (2016) Study 2–Overconfidence Outcome.
