# Supplementary material for: Individual Responses versus Aggregate Group-Level Results: Examining the Strength of Evidence for Growth Mindset Interventions on Academic Performance
Source: J Intell. 2023 May 30;11(6):104. doi: 10.3390/jintelligence11060104 (PMC10299668; doi:10.3390/jintelligence11060104)
Supplement: Supplementary file 1 [file jintelligence-11-00104-s001.zip › jintelligence-2297009-supplementary.pdf]

Supplemental Results

1. Ehrlinger et al. (2016) Study 2 – Overconfidence Outcome

1.1 Introduction

In addition to claims about attention allocation, Ehrlinger et al. made claims about overconfidence. Specifically, they stated that "[t]eaching a growth mindset makes students open to difficulty and less overconfident" (p. 94). They also stated that "[p]articipants who were randomly assigned to a condition in which they were taught an entity (vs. incremental) view of intelligence subsequently allocated less time to difficult problems and, as a result, made more overconfident assessments of performance" (p. 98).

1.2 Methods

To assess the strength of the evidence that students taught a fixed mindset were more overconfident than students taught a growth mindset, we examined each of the fixed mindset condition-growth mindset condition pairs (2,193 in total) and assessed the percentage of pairs in which the student in the fixed mindset condition was more overconfident in their performance than their growth mindset counterpart, the percentage of pairs that were equally overconfident, and the percentage of pairs in which the student in the fixed mindset condition was less (over)confident than their growth mindset counterpart. Overconfidence was determined by subtracting an individual's actual percentile score from their estimated percentile. Higher and positive difference scores would indicate a greater degree of overconfidence, while negative difference scores would indicate an absence of overconfidence.

1.3 Results and Discussion

To assess the claim that holding a fixed mindset promotes greater overconfidence than holding a growth mindset, we examined how often students in the fixed mindset condition were more overconfident in their performance than students in the growth mindset condition. Ehrlinger et al. (2016) reported that participants in the fixed mindset article condition produced more overconfident estimates of their percentile ratings, $p < .005$, $\eta_p^2 = .09$. The results of this individual-level analysis seem to offer some support for the claim that those who endorse a fixed mindset exhibit more overconfidence than those taught a growth mindset. Of the 2,193 fixed mindset-growth mindset student pairs, 64.61% of cases behaved according to claim; that is, the student in the fixed mindset condition was more overconfident in their percentile estimates than their counterpart from the growth mindset condition. In 0.55% of the pairs, overconfidence difference scores were identical. In 34.84% of the pairs, the student in the fixed mindset condition was less overconfident than their counterpart from the growth mindset condition. Thus, about two-thirds of the pairs behave in line with Ehrlinger et al.'s claim and about one-third of the pairs behave counter to claim, where the student taught a growth mindset is more overconfident than their fixed mindset counterpart.
